# Surveillance of pneumococcal diseases in Central and Eastern Europe

**DOI:** 10.1080/21645515.2016.1159363

**Published:** 2016-04-20

**Authors:** Mehmet Ceyhan, Ron Dagan, Abdullah Sayiner, Liudmyla Chernyshova, Ener Çağrı Dinleyici, Waleria Hryniewicz, Andrea Kulcsár, Lucia Mad'arová, Petr Pazdiora, Sergey Sidorenko, Anca Streinu-Cercel, Arjana Tambić-Andrašević, Lyazzat Yeraliyeva

**Affiliations:** a Department of Pediatric Infectious Diseases, School of Medicine, Hacettepe University, Ankara, Turkey; b Pediatric Infectious Disease Unit, Ben-Gurion University of the Negev, Beer-Sheva, Israel; c Department of Chest Diseases, Ege University Faculty of Medicine, Izmir, Turkey; d Department of Pediatric Infectious Diseases and Immunology, National Medical Academy for Postgraduate Education, Kiev, Ukraine; e Eskisehir Osmangazi University Faculty of Medicine, Eskisehir, Turkey; f National Medicines Institute, Division of Clinical Microbiology and Infection Prevention, Warsaw, Poland; g Department of Infectology, Joint Hospital Saint László and Saint István, Budapest, Hungary; h National Reference Centre for Pneumococcal and Haemophilus Diseases, Regional Authority of Public Health, Banská Bystrica, Slovak Republic; i Department of Epidemiology, Charles University Faculty Hospital, Pilsen, Czech Republic; j Research Institute of Children's Infection, St. Petersburg, Russia; k Carol Davila University of Medicine and Pharmacy, Bucharest, Romania; l Department of Clinical Microbiology, University Hospital for Infectious Diseases, Zagreb, Croatia; m Research Institute of Fundamental and Applied Medicine, Asfendiyarov Kazakh National Medical University, Almaty, Kazakhstan

**Keywords:** burden, central Europe, Eastern Europe, epidemiology, mortality, pneumococcal diseases, pneumococcal conjugate vaccines, surveillance, vaccination

## Abstract

Pneumococcal infection is a major cause of morbidity and mortality worldwide. The burden of disease associated with *S. pneumoniae* is largely preventable through routine vaccination. Pneumococcal conjugate vaccines (e.g. PCV7, PCV13) provide protection from invasive pneumococcal disease as well as non-invasive infection (pneumonia, acute otitis media), and decrease vaccine-type nasopharyngeal colonisation, thus reducing transmission to unvaccinated individuals. PCVs have also been shown to reduce the incidence of antibiotic-resistant pneumococcal disease. Surveillance for pneumococcal disease is important to understand local epidemiology, serotype distribution and antibiotic resistance rates. Surveillance systems also help to inform policy development, including vaccine recommendations, and monitor the impact of pneumococcal vaccination. National pneumococcal surveillance systems exist in a number of countries in Central and Eastern Europe (such as Croatia, Czech Republic, Hungary, Poland, Romania and Slovakia), and some have introduced PCVs (Czech Republic, Hungary, Kazakhstan, Russia, Slovakia and Turkey). Those countries without established programs (such as Kazakhstan, Russia and Ukraine) may be able to learn from the experiences of those with national surveillance systems. The serotype distributions and impact of PCV13 on pediatric pneumococcal diseases are relatively similar in different parts of the world, suggesting that approaches to vaccination used elsewhere are also likely to be effective in Central and Eastern Europe. This article briefly reviews the epidemiology of pneumococcal disease, presents the latest surveillance data from Central and Eastern Europe, and discusses any similarities and differences in these data as well the potential implications for vaccination policies in the region.

## Introduction

Pneumococcal disease refers to a wide range of infections caused by the bacterium *Streptococcus pneumoniae* (*S. pneumoniae*), also known as pneumococcus. According to the World Health Organization, pneumococcal diseases (meningitis, bacteraemic and non-invasive pneumonia, and bacteraemia), are the leading cause of vaccine-preventable deaths worldwide among children aged <5 years.^1,2^ The most common clinical presentation in children is pneumonia, which is mostly without bacteraemia. *S. pneumoniae* is also the leading cause of bacterial meningitis among children aged <5 years. In adults, pneumonia is again the most common manifestation of pneumococcal disease.^3^ Mortality due to invasive pneumococcal diseases (IPD) remains high in adult populations, particularly in those aged ≥65 years.^4^

Most pneumococci are encapsulated, their surfaces composed of complex polysaccharides. The polysaccharide capsule is an important determinant of the pathogenicity of pneumococci and forms the basis for classification by serotypes.^3^ While different pneumococcal serotypes vary in their propensity to cause asymptomatic colonisation and invasive disease, only a limited number of serotypes are responsible for most cases of IPD worldwide. The predominant invasive pneumococcal serotypes range across age groups and geographic areas.^3^

Pneumococcal vaccines are targeted primarily at the polysaccharide capsule. The pneumococcal polysaccharide vaccine (PPV) consists of purified polysaccharides from a number of frequent or virulent capsular types of *S. pneumoniae*.^3^ For example, PPV23 provides protection against IPD due to 23 serotypes in children aged ≥2 years and adults.^5^ The newer pneumococcal conjugate vaccines (PCVs) contain capsular polysaccharides from prevalent serotypes conjugated to a protein carrier.^3^ Conjugate vaccines are immunogenic in infants and young children aged < 2 years, and may provide longer lasting immunity against invasive disease compared to PPV.^3,6^ In contrast to PPV, PCV has also been shown to have mucosal impact in young children.^7^ This provides additional benefit by preventing nasopharyngeal carriage of vaccine-type pneumococci and non-invasive pneumococcal diseases, which cause significant morbidity (acute otitis media [AOM] and non-invasive pneumonia) and mortality (non-invasive pneumonia) compared to IPD in children.^7^

National pneumococcal policies vary across regions, ranging from licensure of a single pneumococcal vaccine to the implementation of national PCV programmes. Such policies and recommendations may be influenced by the disease burden and cost effectiveness of alternative interventions in specific countries.^8^ Surveillance systems are critical for monitoring the coverage, impact and effectiveness of vaccination, as well as understanding local pneumococcal epidemiology to guide future vaccination policies and programmes. These data may also help to inform national decision making for local vaccination policy in countries without established programmes. At present, surveillance for IPD is very heterogeneous and many countries lack surveillance systems to determine accurate, up-to-date national estimates of pneumococcal disease burden and have limited data on serotype distribution.

Pneumococcal surveillance data from a number of countries in Central and Eastern Europe together with Kazakhstan were presented at the 7^th^ Pneumo Surveillance Summit, which took place in Istanbul in September 2014. This article reviews the epidemiology of pneumococcal disease, presents the latest pneumococcal surveillance data from Central and Eastern Europe, and discusses any similarities and differences in these data, as well as the potential implications for vaccination policies in the region.

## Epidemiology of pneumococcal disease and influencing factors in children

### Epidemiology, serotype distribution and antibiotic resistance

The highest incidence of IPD is seen in young children aged < 2 years and the elderly.[8] In the majority of cases, pneumococcal disease is preceded by asymptomatic nasopharyngeal colonisation, which is especially high in children.^9^ The global prevalence of *S. pneumoniae* colonisation is estimated at 10% to 85% in children < 5 years of age, and 4% to 45% in adults.^10-12^

While more than 90 different capsular structures/serotypes have been identified, they vary in their propensity to cause asymptomatic colonisation and non-invasive or invasive disease.^13-16^ IPD is more likely to occur from new acquisition of a particular serotype rather than from long-term carriage.^17^ Serotype distribution varies across age groups and geographic areas and may be influenced by vaccination programmes, antibiotic use, socioeconomic conditions and an aging population.^18-21^ In 2011, the serotypes most commonly isolated from IPD cases in Europe were 7F, 19A, 3, 1, 22F, 8, 14, 12F, 6C and 4. For children aged < 1 year, 19A was the most common serotype followed by 7F. In older children aged 1–4 years, serotypes 19A and 1 were most frequently isolated.^22^

Antibiotic resistance in pneumococci is an increasing challenge that impacts on the ability to successfully treat pneumococcal diseases.^23^ Globally, most antibiotic resistance is found in serotypes 6A, 6B, 9V, 14, 15A, 19A, 19F and 23F, although resistance rates also vary geographically.^24^ PCV vaccination has been shown to reduce pneumococcal resistance in vaccinated and unvaccinated populations by reducing the carriage of antibiotic-resistant serotypes and through an overall reduction in antibiotic use.^25^ The introduction of PCV7, the first pneumococcal conjugate vaccine, was associated with a reduction in the total number of resistant isolates.^25^ However, this also led to changes in the pattern of non-PCV7 serotypes causing pneumococcal disease, with an increase in resistant serotype 19A.^26^ Although resistant 19A decreased following the replacement of PCV7 with PCV13 (including 19A antigen), lower IPD rates with PCV13 have also been partially offset by the emergence of drug-resistant pneumococcal strains of non-vaccine serotypes.^27,28^

### Impact of PCV7/13 vaccination on pediatric disease

Pneumococcal conjugate vaccination induces a robust immune response in children < 2 years of age, protecting them against pneumococcal infection as well as reducing asymptomatic nasopharyngeal carriage (colonization) by the specific vaccine serotypes.^6^ The extent and duration of protection provided by PCV is suggested to be greater than PPV due to enhanced immunogenicity by coupling of pneumococcal polysaccharides to carrier proteins.^6^

The introduction of PCV has been associated with significant reductions in the incidence of IPD as well as non-invasive pneumococcal infections such as AOM and pneumonia. In Israel, there was a 63% reduction in all-serotype IPD episodes in children aged < 5 years following the introduction of PCV7, and then PCV13.^29^ The incidence of otitis media enriched with complex cases also fell by 78% after the introduction of PCV7 and PCV13.^30^ With the inclusion of six additional serotypes, including 19A, PCV13 has been shown to result in greater reductions in IPD, AOM and community-acquired pneumonia (CAP) rates compared to PCV7.^30,31^ For example, within 2 years of the introduction of PCV13 for vaccination of infants in the US, there was a 100% reduction in the number of IPD cases in children aged < 2 years caused by PCV13 serotypes.^32^

### Impact of pediatric PCV vaccination on adult pneumococcal disease

The effect of PCV on pneumococcal carriage in young children, the age group most likely to be colonized, can also produce a herd protection effect in unvaccinated older children and adults.^33,34^ Following the introduction of PCV7 and then PCV13, the overall incidence of IPD declined markedly across the entire population (vaccinated and unvaccinated). This can be attributed to the effect of PCV on nasopharyngeal carriage, thus reducing pneumococcal transmission in the community. For example, within 2 years of the introduction of PCV13 in the US, a 47% reduction in IPD was observed in adults aged ≥65 years.^35^ Similarly, in Denmark, there was a 21% reduction in IPD across the entire population following the introduction of PCV13. IPD-related 30-day mortality was estimated to decrease by 28%, mainly due to a decline in mortality in the unvaccinated population.^36^

Despite the herd protection effect of pediatric vaccination on older, unvaccinated populations, the burden of pneumococcal disease in adults remains high. In 2011, the most frequently isolated serotype from IPD cases among adults aged 15–64 years in Europe was 7F; serotypes 3 and 19A were most common in those aged ≥65 years.^22^ Although these serotypes are covered by PCV13, recent data from Spain indicate that PCV13 serotypes are among significant causes of CAP in people aged >50 years.^37^ In the US, declines in the proportion of CAP caused by PCV13 serotypes were found to be significant only among adults living with children.^38^ Adult vaccination with PCV13 may therefore help to reduce the current burden of pneumococcal disease in this population, as shown by the results from the CAPiTA (Community Acquired Pneumonia Immunization Trial in Adults) study ^39^ showing that PCV13 can prevent a significant proportion of pneumococcal CAP in adults aged ≥65 years. In this large-scale (>80,000 subjects), randomized, placebo-controlled efficacy study, patients receiving PCV13 experienced 45% fewer first episodes of non-invasive vaccine-type CAP and 75% fewer first episodes of IPD.^39^ In September 2014, the US. Advisory Committee on Immunization Practices (ACIP) recommended PCV13 for routine use to help protect older adults aged ≥65 years against pneumococcal disease.^40^ The EU license was extended in February 2015 for active immunisation for the prevention of pneumonia caused by *S. pneumoniae* in adults aged ≥18 years and the elderly.^6^ A number of EU countries recommend PCV vaccination for all adults aged >50 years, while others only recommend it in high-risk groups such as immunocompromised patients and those with chronic diseases.

Serotype distribution of pneumococcal disease is more diverse in adults than in children. The unconjugated pneumococcal polysaccharide vaccine, PPV23, which contains more serotypes, is frequently used for pneumococcal vaccination of adults. However, there is strong evidence that PPV is not effective in preventing non-invasive pneumococcal disease. A 2009 meta-analysis of clinical trials of PPV in adults failed to show a protective effect against pneumococcal pneumonia,^41^ and these findings are further supported by a recent Israeli analysis.^42^ While PPV induces significant immune responses in immunocompetent adults, including the elderly, poor responses have been observed in immunocompromised patients. The effect of PCV on nasopharyngeal colonisation may therefore be beneficial for immunocompromised hosts.^43^ In adults aged >50 years, PCV13 has been shown to elicit a greater immune response compared to PPV for the majority of serotypes common to both vaccines, without an associated risk of hyporesponsiveness.^44^ However, there are currently no comparative data demonstrating that PCV provides better protection than PPV in adults.

## Pneumococcal disease in central and eastern european countries

### National surveillance systems, vaccination policies and rates

Surveillance for invasive pneumococcal disease is important to understand local epidemiology and serotype distribution, and monitor the impact of pneumococcal vaccination, including any additional herd protection conferred to unvaccinated individuals and the incidence of disease caused by non-vaccine serotypes (i.e. replacement disease).

A number of countries in Central and Eastern Europe for which data were presented at the 7^th^ Pneumo Surveillance Summit have established national pneumococcal surveillance systems (Table 1). For example, a surveillance database has been operating in Czech Republic since 2008. Combining data from the National Reference Laboratory for Streptococcal Infections and EPIDAT, 82.9% of *S. pneumoniae* strains were serotyped in 2012 and 85.4% in 2013.^45^ In Poland, BINeT was established in 2008 to enhance surveillance carried out since 1997 by the National Reference Center for bacterial infections of the central nervous system; around 180 hospital laboratories are currently involved.^46^ In Slovakia, IPD surveillance began in 1997 with regular serotyping being carried out since 2011,^47^ and in Romania, national surveillance for IPD and serotypes has been in practice since 2010.^48^ There is currently no national IPD surveillance program in Russia as only meningitis is a reportable disease; however, data from three regional programs have been published.^49-51^ In Turkey, national pneumococcal surveillance is not yet established; serotype distribution data have mostly been published from sub-national surveillance and epidemiological research studies run by experts.^52,53^

**Table 1.** Surveillance systems and vaccination policies in the different countries.

	Pneumococcal surveillance system		National immunisation program	
Country	System	Date established	PCV implemented	Date introduced
Croatia	National surveillance on antibiotic resistance	1996	PCV7, PCV10/13	Children aged 2 months to 5 years: 2007 Adults: 2014
	EARSS/EARS-Net	2001		
Czech Republic	Surveillance database combining data from the National Reference Laboratory for Streptococcal Infections and EPIDAT	2008	PCV7	2010
			PCV10 PCV13	2010
Hungary	National Center for Epidemiology (no IPD data collection)	2008	PCV7	2008
			PCV13	2010
Kazakhstan	No national system	–	PCV13	2010–15
Poland	BiNet	2008	PCV7, *PCV10 & PCV13	2009, 2012
Romania	Health Ministry	2010	N/A	–
Russia	No national system	–	PCV13	2014
Slovakia	National Reference Center for Pneumococcal Diseases	2011	PCV7 PCV13 PCV10	2009 2010 2011
Turkey	Sub-national system	–	PCV7	2008
			PCV13	2011
Ukraine	None	–	*PCV10/13	2011

*Note.* *High-risk groups only

Pneumococcal conjugate vaccines are available in most countries in the region and have been introduced as part of the national immunization program (NIP) in several of the countries discussed in this article (Table 1). In the Czech Republic, pneumococcal vaccination of infants began in 2010. Both PCV10 and PCV13 are available through the NIP; PCV10 is fully covered by insurance while PCV13 requires a co-payment.^45^ PCV7 was introduced as part of the pediatric NIP in Hungary in 2008; PCV13 has been in use since 2010 and has been listed on the mandatory immunization schedule for children aged < 2 years since 2014; the vaccination rate is now almost 100%.^54^ In Turkey, PCV7 was introduced to the NIP in 2008, with a switch to PCV13 in 2011; the vaccination rate is estimated to be more than 95% for children < 2 years of age.^55^ PCV13 was introduced to the Russian NIP in 2014. In Poland, pediatric pneumococcal vaccination is part of the NIP but only for at-risk groups, such as low birth weight and preterm infants. Pediatric pneumococcal vaccination is yet not part of the NIP in Romania and Ukraine.

### Nasopharyngeal carriage

Data on nasopharyngeal carriage rates and the impact of pediatric pneumococcal vaccination on nasopharyngeal colonization are available for several countries in Central and Eastern Europe. In Hungary, the pneumococcal nasopharyngeal (PNP) carriage rate among nasopharyngeal swabs from 227 children aged < 2 years who received PCV13 was found to be 41%. The most prevalent serotypes were 11A/D/F, 15B/C, 35F, 47F and 23B, all of which are non-vaccine serotypes. Those serotypes covered by PCV13 have almost disappeared since the introduction of PCV13 in 2010.^56^ For Ukraine, there are currently limited data available on PNP carriage rates. The overall incidence of *S. pneumoniae* carriage among nasopharyngeal swabs collected from 1,000 healthy children aged 6 months to 5 years between 2012 and 2014 was 50.4%. The most prevalent serotypes were 19F, 6A/B, 14 and 23F, with potential PCV13 serotype coverage estimated to be 47% in infants aged < 1 year and 63% in children aged >4 years.^57^ A study of pediatric PNP carriage in Romania found the incidence of PNP carriage was 25% in 2011–13, with 59% of the serotypes isolated being covered by PCV13.^48^ In Russia, PCV13 was found to cover 78% of isolates collected from nasopharyngeal swabs from children aged < 5 years in Moscow; serotype 19F was more prevalent in children aged < 2 years, and serotype 3 was predominant in those >2 years.^50^

### Invasive pneumococcal disease

Globally, the peak incidence of IPD occurs in early infancy and older adults and this is reflected in data available for three countries in the Central and Eastern European region on the age distribution of IPD (Table 2). In Poland, for example, the peak incidence of IPD in 2011–2013 was in infants aged < 1 year and adults >85 years, although the case fatality rate (CFR) was much higher in older adults.^58^ There is considerable variation in IPD rates reported for different countries in Central and Eastern Europe, with a trend toward an increased incidence of IPD over time. In Slovakia, for example, the IPD incidence is highest in children < 1 year and increased between 1997 and 2013.^59^ In Czech Republic, following the introduction of PCV in 2010, both the total incidence and the incidence in the 0–4-year age group decreased from 2011 to 2012 and increased again in 2013.^60^ Between 2012 and 2013, the incidence of IPD in Czech Republic increased from 1.8 to 9.2/100,000 in infants < year of age, and from 2.7 to 3.8/100,000 in those aged 1–4 years. In the same period, the IPD incidence in older adults also increased from 3.3 to 4.3/100,000 in those aged 40–64 years and 8.1 to 10.2/100,000 in those aged ≥65 years; the CFR in over-65s was 22.2%. Several reasons have been proposed for the increase observed, including improved surveillance, serotype replacement and a decline in vaccination coverage.^45^ In Czech Republic, vaccination rates in infants have declined in the past 2 years to close to 70% (< 50% in Prague). The vaccination rate is estimated at < 0.01% in adults, although it is 80% in nursing homes where vaccination is mandatory.^45^

**Table 2.** IPD incidence per 100,000 in various age groups in the different countries.

		Age group (years)						
Country	Year	<1	1–4	3–5	6–17	≥20	50–64	≥65
Czech Republic	2012^65^	1.8	2.7	–	2.2 (5–9 years), 1.3 (10–14 years), 0.7 (15–19 years)	1.3 (20–39 years)	3.3 (40–64 years)	8.1
	2013^66^	9.2	3.8	–	2.5 (5–9 years), 0.9 (10–14 years), 1.2 (15–19 years)	1.3 (20–39 years)	4.3 (40–64 years)	10.2
Poland^58^	2011	4.64	2.80	2.42	0.77 (5–9 years), 0.42 (10–14 years), 0.22 (15–19 years)	0.29 (20–39 years)	1.34	1.89
	2012	3.17	3.11	3.12	0.91 (5–9 years), 0.27 (10–14 years), 0.09 (15–19 years)	0.23 (20–39 years)	1.18	2.45
	2013	5.01	2.43	1.5	1.02 (5–9 years), 0.59 (10–14 years), 0.18 (15–19 years)	0.45 (20–39 years)	1.57	3.33
Slovakia^68^	2011	6.65	0.44	–	–	–	–	1.98
	2012	7.25	3.5	–	–	–	–	1.73
	2013	10.64	2.12	–	–	–	–	3.37

### Serotype prevalence and coverage by vaccines

The predominant invasive pneumococcal serotypes range across age groups and geographic areas. Data on the prevalence of serotypes causing IPD in infants and adults in Central and Eastern European countries are shown in Tables 3 and 4. Some information is also available on serotype coverage by the available conjugate vaccines in countries in the region (Table 5).Where available, data are presented separately for 2011, 2012 and 2013. Where data have come from different sources and cover different regions and/or years (e.g., for Russia), data have not been combined.

**Table 3.** Serotype distribution in children with IPD in the different countries.

			Serotype														
Country	Year/ region	Age group	1	3	4	5	6A	6B	7F	9A	9V	14	15C	18C	19A	19F	23F
Croatia, n (%)^64^	2011	<5	0	0	0	0	1 (5.9)	3 (17.6)	1 (5.9)	0	0	2 (11.8)	0	2 (11.8)	1 (5.9)	1 (5.9)	1 (5.9)
		5–13	0	0	0	0	0	0	0	0	0	0	0	0	0	0	0
	2012	<5	<5	2 (4.9)	0	1 (2.4)	0	1 (2.4)	5 (12.2)	1 (2.4)	0	0	12 (29.3)	0	2 (4.9)	9 (22)	1 (2.4)
		5–13	2 (66.7)	0	0	0	1 (33.3)	0	0	0	0	0	0	0	0	0	0
	2013	<5	0	0	0	0	1 (2.8)	3 (8.6)	0	0	1 (2.8)	13 (37.1)	0	1 (2.8)	6 (17.1)	0	3 (8.6)
		5–13	1 (20)	0	1 (20)	0	1 (20)	0	0	0	0	1 (20)	0	0	0	0	0
Czech Republic, n (%)^66,67^	2011	<5	1 (4.8)	2 (9.5)	0	0	2 (9.5)	0	1 (4.8)	0	0	2 (9.5)	1 (4.8)	2 (9.5)	2 (9.5)	0	1 (4.8)
	2012		1 (6.7)	0	0	0	1 (6.7)	0	0	0	0	1 (6.7)	1 (6.7)	0	2 (13.3)	1 (6.7)	1 (6.7)
	2013		3 (10.7)	1 (3.6)	0	0	0	0	1 (3.6)	0	1 (3.6)	1 (3.6)	0	0	5 (17.8)	1 (3.6)	0
Hungary, n (%)^69,70^	2010–13	≤5	7 (10.3)	16 (23.5)	0	2 (2.9)	1 (1.5)	2 (2.9)	6 (8.8)	0	1 (1.5)	0	4 (5.9)	2 (2.9)	19 (27.9)	3 (4.4)	3 (4.4)
		6–18	6 (22.2)	2 (7.4)	1 (3.7)	2 (7.4)	1 (3.7)	2 (7.4)	3 (11.1)	0	1 (3.7)	0	0	0	1 (3.7)	2 (7.4)	1 (3.7)
Poland, %^58^	2011–13	≤5	3.8	6.3	3.8	—	2.5	11.3	0.6	—	4.4	18.2	6.9	2.5	9.4	8.2	7.5
Romania, %^71^	2010–13	≤5	2	5	—	1	—	8	—	—	3	4	—	—	5	19	3
		6–17	—	3	—	—	—	—	2	—	—	1	—	—	—	3	—
Russia, n (%)	St Petersburg 2010–2012 [49] (n=49)^#^	<18	0	7	0	0	6	0	5	5	0	3	0	4	5		
	St Petersburg 2010–2012 [49] (n=118) ^#^	<5	0	21	0	0	10	0	1	3	0	1	4	33	10		
	Various regions 2012–13[51] ^*^	<5	0	(11.1)	0	0	(2.3)	(11.1)	(0.5)	(1.4)	(2.3)	(15.8)	(0.5)	(2.8)	(7.0)	(22.9)	(13.5)
	Moscow 2009–13[50] ^†^	IQR 0.1–6.0	3 (0.4)	63 (7.5)			70 (8.4)	107 (12.8)	10 (1.2)	—	13 (1.6)	75 (9.0)	13 (1.6)	20 (2.4)	19 (2.3)	181 (21.7)	84 (10.1)
Slovakia, n (%)^59^	2011–12	≤5	—	1 (5.6)	—	—	—	1 (5.6)	1 (5.6)	—	—	2 (11.1)	—	—	5 (27.8)	2 (11.1)	1 (5.6)
	2013	≤5	—	2 (18.2)	—	—	—	—	—	—	—	1 (9.1)	1 (9.1)	—	3 (27.3)	1 (9)	—
Turkey, n (%)	2008–10^52^	≤5	6 (4.1)	6 (4.1)	11 (7.5)	4 (2.7)	3 (2.1)	14 (9.6)	1 (0.7)	—	1 (0.7)	10 (6.8)	2 (1.4)	4 (2.7)	9 (6.2)	38 (26)	8 (5.5)
		>5–≤18	2 (3.6)	4 (7.1)	3 (5.4)	—	2 (3.6)	2 (3.6)		—	1 (1.8)	2 (3.6)	3 (5.4)	1 (1.8)	1 (1.8)	3 (5.4)	—
	2010–11^72^^**^	<18	8 (14.5)	5 (9.1)	—	7 (12.7)	—	2 (3.6)	1 (1.8)	—	1 (1.8)	2 (3.6)	—		1 (1.8)	3 (5.5)	1 (1.8)

*Note.*

# Community-acquired pneumonia – lytA positive blood samples – PCR typing from blood

* Acute otitis media.

† Acute otitis media + carriage.

** empyema #serotype 15B/C.

**Table 4.** Serotype distribution in adults with IPD in the different countries.

			Serotype																
Country	Year	Age group	1	3	4	5	6A	6B	7F	8	9	10A	11A	14	18	19A	19F	22F	23F
Croatia, n (%)^64^	2011	18–65	3 (10.3)	4 (13.8)	2 (6.9)	0	1 (3.4)	1 (3.4)	2 (6.9)	0	2 (6.9)	2 (6.9)	0	7 (26)	0	0	1 (3.4)	1 (3.4)	0
		>65	2 (6.3)	12 (37.5)	1 (3.1)	0	1 (3.1)	0	0	1 (3.1)	2 (6.3)	1 (3.1)	1 (3.1)	2 (6.3)	2 (6.3)	1 (3.1)	1 (3.1)	0	1 (3.1)
	2012	18–65	5 (10.6)	13 (27.6)	1 (2.1)	0	1 (2.1)	2 (4.2)	1 (2.1)	0	2 (4.2)	0	1 (2.1)	2 (4.2)	1 (2.1)	4 (8.5)	1 (2.1)	0	2 (4.2)
		>65	0	10 (30.3)	1 (3)	0	1 (3)	1 (3)	0	0	3 (9)	1 (3)	4 (12)	0	1 (3)	2 (6)	1 (3)	1 (3)	1 (3)
	2013	18–65	2 (5.9)	10 (29.4)	2 (5.9)	0	2 (5.9)	1 (3)	1 (3)	0	1 (3)	0	1 (3)	6 (17.6)	0	2 (5.9)	1 (3)	0	2 (5.9)
		>65	0	6 (16.2)	2 (5.4)	0	2 (5.4)	1 (2.7)	2 (5.4)	0	4 (10.8)	0	0	9 (24.3)	0	4 (10.8)	1 (2.7)	1 (2.7)	2 (54)
Czech Republic, n (%)^66,67^	2011	≥65	7 (5.9)	18 (15.2)	8 (6.8)	0	2 (1.7)	3 (2.5)	14 (11.9)	2 (1.7)	3 (2.5) (9V); 5 (4.2) (9N)	5 (4.2)	2 (11.7)	8 (6.8)	2 (1.7)	6 (5.1)	2 (1.7)	7 (5.9)	6 (5.1)
	2012		5 (3.6)	20 (14.4)	4 (2.9)	0	3 (2.2)	3 (2.2)	8 (5.8)	3 (2.2)	10 (7.2) (9V); 10 (7.2) (9N)	5 (3.6)	9 (6.5)	8 (5.8)	3 (2.2)	3 (2.2)	0	8 (5.8)	4 (2.9)
	2013		8 (4.4)	28 (15.6)	2 (1.1)	0	5 (2.8)	2 (1.1)	4 (2.2)	6 (3.3)	2 (1.1) (9V); 9 (5.0) (9N)	5 (2.8)	3 (1.7)	10 (5.6)	1 (0.6)	12 (6.7)	2 (1.1)	11 (6.1)	5 (2.8)
Hungary, n (%)^56,74^	2010–13	19–65	7 (2.3)	83 (26.7)	7 (2.3)	0	16 (5.1)	10 (3.2)	15 (4.8)	14 (4.5)	3 (1) (9V); 11 (3.5) (9N)	7 (2.3)	12 (3.9)	5 (1.6)	0	14 (4.5)	10 (3.2)	10 (3.2)	8 (2.6)
		>65	2 (0.9)	76 (34.5)	2 (0.9)	0	9 (4.1)	8 (3.6)	8 (3.6)	10 (4.5)	3 (1.4) (9V); 3 (1.4) (9N)	1 (0.5)	10 (4.5)	4 (1.8)	0	12 (5.5)	9 (4.1)	6 (2.7)	0
Poland, %^58^	2010–13	≥65	3.8	22.2	4.9	–	1.1	1.6	1.9	1.6	3.8 (9N)	1.9	2.2	8.1	–	6.2	5.7	3.0	4.6
Romania, %^71^	2010–13	18–64	2	3	–			5	2					1		1	2		1
		≥65	1	3	–			4	1								2		1
Russia, n ^49^^#^	2010–13		0	6	–		3 (6A/B/C)	0		1	1		2	0	4	4		5	
Slovakia, n (%)^59^	2011–12	>18	0	17 (26.6)	–		3 (4.7)	2 (3.1)	1 (1.6)		2 (3.1)	1 (1.6)		3 (4.7)	1 (1.6)	10 (15.6)	2 (3.1)		0
	2013	>18	3 (5.2)	11 (19.0)	–		0	4 (6.9)	6 (10.3)		1 (1.7)	0		1 (1.7)	0	3 (5.2)	0		1 (1.7)
Turkey, % ^53^	2010–12	–	7.1	17.1	2.8	2.8	7.1	1.4	–	–	1.4	–		5.7	1.4	2.8	12.8	–	1.4
	2005–11	>18	2.9	11.8	1.8	0.7	0.7	5.0	1.8	–	1.4	–	–	4.6	0.4	6.8	12.1	–	2.1

Note.

# Community-acquired pneumonia – lytA positive blood samples – PCR typing from blood.

**Table 5.** Coverage rates of invasive isolates by vaccines in the different countries.

		PCV7		PCV10		PCV13	
Country	Year/region	Paed	Adult	Paed	Adult	Pediatric	Adult
Croatia^64^	2011	52.9%	35.5%	58.8%	46.8%	70.6%	82.3%
	2012	59%	25.6%	70.5%	32.9%	95.5%	72%
	2013	60%	41.6%	62.5%	48%	82.5%	76.7%
Czech Republic^66-68^	2011	23.8%^#^	27.1^**^	33.3%^#^	44.9%^**^	61.9%^#^	66.9%^**^
	2012 (including non-typed)	20.0%^#^	21.0^**^	26.7%^#^	23.0%^**^	46.7%^#^	51.1%^**^
	2013 (including non-typed)	10.7%^#^	13.3%^**^	25.0%^#^	20.0%^**^	46.4%^#^	45.0%^**^
	2014 (including non-typed)	20.0%^#^	11.7^**^	32.0%^#^	19.5%^**^	52.0%^#^	46.8%^**^
Hungary^56,73^	2013	5%	12.4%^‡^	10%	16.8%^‡^	50%	56.6%^‡^
Poland^58^	2011–13	–	60.4%^#^	38.3^**^%	78.6%^#^	68.7^**^%	
Romania^72^	2010–13	59.8%	72.7%	97.4%			
Russia	St Petersburg 2010–2012 ^49^	49.2%	–	49.2%	65%	70.4%	95%
	Various regions 2012–13^51^	68.4%^#^	–	68.9%^#^	–	87.0%^#^	–
	Moscow 2009–13 ^50^	62.8%^*^	–	64.5%^*^	–	81.2%^*^	–
Slovakia^59^	2011–13	27.6%	18%^**^	31%	18%^**^	69%	64.1%^**^
Turkey	1998–2007^74^	45.3%	40.2%	87.3%	75.5%	92.1%	85.5%
	2001–04^75^	63%^*^	–	–	77.8%^*^	–	
	2008–10^52^	69.5%^*^	–	75.8%^*^	–	85.3%^*^	–
	1996–2008^76^	44.1%	39.8%	–	66.1%	71.5%	
	2005–11^53^	–	–	–	–	49.4%	
	2010–11^72^	16.3%^†^	–	45.4%^†^	–	60%^†^	–
	2011–12^77^	48%^#^	–	51%^#^	71%^#^	–	

*Note.* Pediatric ≤5 years unless otherwise specified. Adult >18 years unless otherwise specified.

* ≤2 years.

† ≤18 years.

‡ ≥60 years.

# <5 years

** adults ≥65 years.

Between 2005 and 2014, the most common serotypes among invasive pneumococcal isolates collected from children aged < 5 years in Croatia were 14, 19A, 6B, 23F and 18C. The serotype prevalence in adults was found to be more diverse, with the most common serotypes being 3, 14, 1, 19A, 7F, 9 and 23F.^61^ Serotype prevalence data from Czech Republic in 2012–13 show 19A and 1 were the most frequent invasive serotypes isolated from children aged < 5 years, and 3, 9, 22F, 14 and 19A for older adults aged ≥65 years.^45^ Vaccine serotype coverage rates with PCV13 are 88% for pediatric patients and 80% for adults in Croatia,^61^ and 46% and 45%, respectively, in Czech Republic.^45^ In Poland, serotypes 3 and 14 were responsible for most IPD cases from 2011–13, however, serotype 11A caused the highest CFR.^46^ PCV10 and PCV13 were reported to cover 46.0% and 71.8% of all IPD cases; 61.4% and 79.5% of cases in children aged < 2 years; and 60.4% and 78.6% of cases involving children < 5 years of age, respectively. PCV13 and PPV23 covered 68.7% and 86.0% of cases in adults aged >65 years, respectively.^46^ In Romania, IPD cases are currently under reported and there are limited data on serotype distribution for different age groups. Among a collection of 360 isolates from IPD across all age groups, only 90 isolates were serotyped; 19F was the most prevalent serotype in children aged ≤ 5 years, followed by 6B, 19A and 3. Between 2010 and 2013, the potential vaccine coverage rate of invasive serotypes in Romania was estimated at 73% for PCV10 and 97% for PCV13.^48^

Following the introduction of PCVs in Central and Eastern Europe, changes have been observed in the distribution of invasive serotypes, with the emergence of non-vaccine serotypes reported in some countries. In Slovakia, the most prevalent serotypes in pediatric age groups before the introduction of PCV7 in 2007 were 14 and 19A, shifting to 19A and 3 in 2013. In adults, serotypes 3 and 19A were most frequent in 2011–12, with 7F and 6B increasing in incidence in 2013.^47^ In Turkey, PCV7 was introduced in 2008 and the most common IPD serotypes in children ≤ 2 years in 2008–10 were 19F and 6B.^52^ In 2011–12, before the switch to PCV13, the serotype distribution had changed, with serotypes 3, 6A and 19A being the most prevalent.^62^

### Antibiotic resistance

Recent antibiotic use is strongly associated with carriage of resistant pneumococci. Among individuals who develop IPD, recent antibiotic use is also associated with an increased risk of infection with a resistant strain. Several countries in Central and Eastern Europe have reported on the frequency of local antibiotic resistance and non-susceptibility among *S. pneumoniae* isolates. In Croatia, the proportion of penicillin-non-susceptible invasive isolates was 17% in 2005 and 23% in 2012,^63^ with serotypes 14 and 19A accounting for the highest number of non-susceptible isolates.^64^ All highly resistant isolates appear to have been prevented by the introduction of PCV13. High rates of resistance to macrolides and penicillin have been observed in Romania, especially in serotypes covered by PCV13.^48^ Antibiotic resistance rates among nasopharyngeal isolates in Hungary have been reported at 22% for erythromycin and 17% for clindamycin and tetracycline; 79% of isolates were sensitive to penicillin and 21% had intermediate resistance.^56^ Nasopharyngeal isolates in Ukraine have shown resistance to ciprofloxacin (100%), co-trimoxazole (48%), erythromycin (33%), azithromycin (33%), amoxicillin/clavulanate (R and I, 33%), penicillin (20%) and cefuroxime (12%). Thirty-five percent were multi-drug resistant, with serotypes 14, 19F, 6A/B and 23F being the most resistant.^57^ Between 60% and 80% of isolates from IPD cases in Poland were susceptible to penicillin across the different age groups, with ˜55–75% susceptible to erythromycin, >80% susceptible to cefotaxime and >90% susceptible to chloramphenicol and meropenem. The highest non-susceptibility rates were in the youngest patients. Serotypes 19A, 19F and 6B were the most multi-drug resistant. PCV13 and PPV23 each covered >90% of multi-drug resistant serotypes, compared with only ˜65% for PCV10.^46^ Antibiotic resistance has increased in recent years in Russia, with macrolide non-susceptibility rates of 26% and 31% reported in Moscow and St Petersburg, respectively.^49,50^ In Turkey, penicillin resistance was found in 23% of isolates and erythromycin resistance in 25% across eight cities; 19A and 19F had the highest resistance rates.^53^ These serotypes are covered by PCV13 which is currently in use.

## Discussion

The data brought together here demonstrate the progress that has been made in Central and Eastern Europe in recent years in terms of pneumococcal surveillance and vaccination. Most of the countries represented now have national surveillance systems and several of them have introduced vaccination with PCVs. However, further efforts are needed to ensure that this progress continues. Pneumococcal surveillance improves understanding of local pneumococcal epidemiology (e.g. serotype distribution and the prevalence of antibiotic resistance), which can help to inform future vaccination policies and programs. Countries without established surveillance systems may be able to learn from the experience of those with established systems. The data from Kazakhstan (Box 1) demonstrate how vaccination can reduce pneumococcal disease and help to reduce pediatric pneumonia rates, which is a major factor contributing to infant and child mortality in those aged <5 years.

**Box 1 Kazakhstan case study.^65^**

There is no established surveillance system in Kazakhstan, and no routine serotyping of isolates. Pneumonia caused almost a third of deaths in infants aged <1 year in 2008, and *S. pneumoniae* was responsible for almost half of the cases of non-meningococcal meningitis in children between 1993 and 2007.

Pneumococcal vaccination of newborns has been implemented on a region-by-region basis since 2010, starting in East Kazakhstan and Mangystau regions. Between 2010 and 2012, the incidence of pneumonia in infants <1 year of age fell from 34.5 to 18.6/1,000 in East Kazakhstan, and from 29.2 to 14.7/1,000 in Mangystau. In Almaty, where vaccination is due to start in 2015, the incidence was 49.4/1,000 in 2010 and 46.2/1,000 in 2012. Infant mortality (before 1 year of age) from pneumonia fell from 10.93 to 4.69/10,000 live births in Mangystau and 13.87 to 11.72/10,000 in East Kazakhstan regions between 2010 and 2012.

A pilot study of pneumococcal epidemiology in children aged <5 years began in 2013 in two cities.

For the countries with serotype data, the majority of serotypes are covered by PCV13. The most common serotypes in children are 6B, 14, 19A and 23F, which are all covered by PCV13. As seen in EU/EEA countries as a whole,^22^ serotypes 1 and 3 are relatively more common in adult patients than in children. Overall, serotype 14 appears to be reported more frequently, and7F and 22F less frequently, in Central and Eastern Europe compared with EU/EEA countries overall.^22^ The serotype coverage by PCV13 of around 50–90% are comparable to those reported for the EU/EEA overall.^22^ In general, the serotype distributions and impact of PCV13 on different forms of pediatric pneumococcal diseases are relatively similar in different parts of the world, suggesting that approaches to vaccination used elsewhere are also likely to be effective in Central and Eastern Europe.

## References

[1] Black RE, Cousens S, Johnson HL, Lawn JE, Rudan I, Bassani DG, Jha P, Campbell H, Walker CF, Cibulskis R, et al. Global, regional, and national causes of child mortality in 2008: a systematic analysis. Lancet 2010; 375:1969-87; PMID:20466419; http://dx.doi.org/10.1016/S0140-6736(10)60549-1 20466419

[2] O'Brien KL, Wolfson LJ, Watt JP, Henkle E, Deloria-Knoll M, McCall N, Lee E, Mulholland K, Levine OS, Cherian T Burden of disease caused by Streptococcus pneumoniae in children younger than 5 years: global estimates. Lancet 2009; 374:893-902; http://dx.doi.org/10.1016/S0140-6736(09)61204-6 19748398

[3] Centers for Disease Control and Prevention Epidemiology and Prevention of Vaccine-Preventable Diseases, 13th edn. 2015. Available at: www.cdc.gov/vaccines/pubs/pinkbook/downloads/pneumo.pdf

[4] Ludwig E, Bonanni P, Rohde G, Sayiner A, Torres A. The remaining challenges of pneumococcal disease in adults. Eur Respir Rev 2012; 21:57-65; PMID:22379175; http://dx.doi.org/10.1183/09059180.00008911 22379175 PMC9487474

[5] PNEUMOVAX® 23 (pneumococcal vaccine polyvalent) US Prescribing Information.

[6] Prevenar 13® (pneumococcal 13-valent conjugate vaccine) Summary of Product Characteristics Kent, UK: Pfizer Ltd

[7] Veenhoven RH, Bogaert D, Schilder AGM, Rijkers GT, Uiterwaal CSPM, Kiezebrink HH, van Kempen MJ, Dhooge IJ, Bruin J, Ijzerman EP, et al. Nasopharyngeal pneumococcal carriage after combined pneumococcal conjugate and polysaccharide vaccination in children with a history of recurrent acute otitis media. Clin Infect Dis 2004; 39:911-9; PMID:15472839; http://dx.doi.org/10.1086/422651 15472839

[8] Pebody RG, Leino T, Nohynek H, Hellenbrand W, Salmaso S, Ruutu P Pneumococcal vaccination policy in Europe. Eurosurveill 2005; 10(9):174-8. 16280609

[9] Bogaert D, de Groot R, Hermans PWM. Streptococcus pneumoniae colonisation: the key to pneumococcal disease. Lancet Infect Dis 2004; 4(3):144-54; PMID:14998500; http://dx.doi.org/10.1016/S1473-3099(04)00938-7 14998500

[10] Cardozo DM, Nascimento-Carvalho CM, Souza FR, Silva NM. Nasopharyngeal colonization and penicillin resistance among pneumococcal strains: a worldwide 2004 update. Braz J Infect Dis 2006; 10:293-303; PMID:17293914; http://dx.doi.org/10.1590/S1413-86702006000400015 17293914

[11] Regev-Yochay G, Raz M, Dagan R, Porat N, Shainberg B, Pinco E, Keller N, Rubinstein E. Nasopharyngeal carriage of Streptococcus pneumoniae by adults and children in community and family settings. Clin Infect Dis 2004; 38:632-39; PMID:14986245; http://dx.doi.org/10.1086/381547 14986245

[12] Chi DH, Hendley JO, French P, Arango P, Hayden FG, Winther B. Nasopharyngeal reservoir of bacterial otitis media and sinusitis pathogens in adults during wellness and viral respiratory illness. Am J Rhinol 2003; 17(4):209-14; PMID:12962190 12962190

[13] Sandgren A, Sjöström K, Olsson-Liljequist B, Christensson B, Samuelsson A, Kronvall G, Henriques Normark B. Effect of clonal and serotype-specific properties on the invasive capacity of Streptococcus pneumoniae. J Infect Dis 2004; 189:785-96; PMID:14976594; http://dx.doi.org/10.1086/381686 14976594

[14] Feldman C, Anderson R. Recent advances in our understanding of Streptococcus pneumoniae infection. F1000Prime Rep 2014; 6:82; PMID:25343039; http://dx.doi.org/10.12703/P6-82 25343039 PMC4166932

[15] Song JY, Nahm MH, Moseley MA. Clinical implications of pneumococcal serotypes: invasive disease potential, clinical presentations, and antibiotic resistance. J Korean Med Sci 2013; 28:4-15; PMID:23341706; http://dx.doi.org/10.3346/jkms.2013.28.1.4 23341706 PMC3546102

[16] Weinberger DM, Harboe ZB, Sanders EAM, Ndiritu M, Klugman KP, Ruckinger S, Dagan R, Adegbola R, Cutts F, Johnson HL, et al. Association of serotype with risk of death due to pneumococcal pneumonia: a meta-analysis. Clin Infect Dis 2010; 51:692-9; PMID:20715907; http://dx.doi.org/10.1086/655828 20715907 PMC2927802

[17] Ferreira DM, Jambo KC, Gordon SB. Experimental human pneumococcal carriage models for vaccine research. Trends Microbiol 2011; 19:464-70; PMID:21784641; http://dx.doi.org/10.1016/j.tim.2011.06.003 21784641

[18] Feikin DR, Klugman KP. Historical changes in pneumococcal serogroup distribution: implications for the era of pneumococcal conjugate vaccines. Clin Infect Dis 2002; 35:547-55; PMID:12173128; http://dx.doi.org/10.1086/341896 12173128

[19] Feikin DR, Klugman KP, Facklam RR, Zell ER, Schuchat A, Whitney CG. Increased prevalence of pediatric pneumococcal serotypes in elderly adults. Clin Infect Dis 2005; 41:481-7; PMID:16028155; http://dx.doi.org/10.1086/432015 16028155

[20] Guevara M, Barricarte A, Gil-Setas A, Garcia-Irure JJ, Beristain X, Torroba L, Petit A, Polo Vigas ME, Aguinaga A, Castilla J. Changing epidemiology of invasive pneumococcal disease following increased coverage with the heptavalent conjugate vaccine in Navarre, Spain. Clin Microbiol Infect 2009; 15:1013-9; PMID:19673968; http://dx.doi.org/10.1111/j.1469-0691.2009.02904.x 19673968

[21] Kellner JD, Vanderkooi OG, MacDonald J, Church DL, Tyrrell GJ, Scheifele DW. Changing epidemiology of invasive pneumococcal disease in Canada, 1998-2007: update from the Calgary-area Streptococcus pneumoniae research (CASPER) study. Clin Infect Dis 2009; 49:205-12; PMID:19508165; http://dx.doi.org/10.1086/599827 19508165

[22] European Centre for Disease Prevention and Control Surveillance of invasive bacterial diseases in Europe, 2011 Stockholm: ECDC, 2013.

[23] Improving global health by preventing pneumococcal disease Report from the All-Party Parliamentary Group on Pneumococcal Disease Prevention in the Developing World. Available at: www.gavi.org/Library/Documents/AMC/All-Party-Parlimentary-Group-on-Pneumococcal-Disease-Report

[24] Felmingham D, Cantón R, Jenkins SG. Regional trends in β-lactam, macrolide, fluoroquinolone and telithromycin resistance among Streptococcus pneumoniae isolates 2001-2004. J Infect 2007; 55:111-8; PMID:17568680; http://dx.doi.org/10.1016/j.jinf.2007.04.006 17568680

[25] Dagan R, Klugman KP. Impact of conjugate pneumococcal vaccines on antibiotic resistance. Lancet Infect Dis 2008; 8(12):785-95; PMID:19022193; http://dx.doi.org/10.1016/S1473-3099(08)70281-0 19022193

[26] Fenoll A, Granizo JJ, Aguilar L, Giménez MJ, Aragoneses-Fenoll L, Hanquet G, Casal J, Tarragó D. Temporal trends of invasive Streptococcus pneumoniae serotypes and antimicrobial resistance patterns in Spain from 1979 to 2007. J Clin Microbiol 2009; 47:1012-20; PMID:19225097; http://dx.doi.org/10.1128/JCM.01454-08 19225097 PMC2668361

[27] Muñoz-Almagro C, Esteva C, Fernandez de Sevilla M, Selva L, Gene A, Pallares R Emergence of invasive pneumococcal disease caused by multidrug-resistant serotype 19A among children in Barcelona. J Infect 2009; 59:75-82; http://dx.doi.org/10.1016/j.jinf.2009.05.012 19576637

[28] McElligott M, Vickers I, Carrferkey M, Cunney R, Humphreys H. Non-invasive pneumococcal serotypes and antimicrobial susceptibilities in a paediatric hospital in the era of conjugate vaccines. Vaccine 2014; 32:3495-500; PMID:24795223; http://dx.doi.org/10.1016/j.vaccine.2014.04.047 24795223

[29] Ben-Shimol S, Greenberg D, Givon-Lavi N, Schlesinger Y, Somekh E, Aviner S, Miron D, Dagan R. Early impact of sequential introduction of 7-valent and 13-valent pneumococcal conjugate vaccine on IPD in Israeli children. Vaccine 2014; 32:3452-9; PMID:24690148; http://dx.doi.org/10.1016/j.vaccine.2014.03.065 24690148

[30] Ben-Shimol S, Givon-Lavi N, Leibovitz E, Raiz S, Greenberg D, Dagan R. Near-elimination of otitis media caused by 13-valent pneumococcal conjugate vaccine (PCV) serotypes in southern Israel shortly after sequential introduction of 7-valent/13-valent PCV. Clin Infect Dis 2014; 59:1724-32; PMID:25159581; http://dx.doi.org/10.1093/cid/ciu683 25159581

[31] Angoulvant F, Levy C, Grimprel E, Varon E, Lorrot M, Biscardi S, Minodier P, Dommergues MA, Hees L, Gillet Y, et al. Early impact of 13-valent pneumococcal conjugate vaccine on community-acquired pneumonia in children. Clin Infect Dis 2014; 58:918-24; PMID:24532543; http://dx.doi.org/10.1093/cid/ciu006 24532543

[32] Steens A, Bergsaker MA, Aaberge IS, Rønning K, Vestrheim DF. Prompt effect of replacing the 7-valent pneumococcal conjugate vaccine with the 13-valent vaccine on the epidemiology of invasive pneumococcal disease in Norway. Vaccine 2013; 31:6232-8; PMID:24176490; http://dx.doi.org/10.1016/j.vaccine.2013.10.032 24176490

[33] Azzari C, Resti M. Reduction of carriage and transmission of Streptococcus pneumoniae: the beneficial “side effect” of pneumococcal conjugate vaccine. Clin Infect Dis 2008; 47:997-9; PMID:18781870; http://dx.doi.org/10.1086/591967 18781870

[34] Simonsen L, Taylor RJ, Young-Xu Y, Haber M, May L, Klugman KP. Impact of pneumococcal conjugate vaccination of infants on pneumonia and influenza hospitalization and mortality in all age groups in the United States. MBio 2011; 2:e00309-10; PMID:21264063; http://dx.doi.org/10.1128/mBio.00309-10 21264063 PMC3025524

[35] Moore MR. Update on effectiveness and impact of PCV13 use among US children Presented at the Advisory Committee on Immunization Practices (ACIP), February 20–21, 2013, Atlanta, Georgia.

[36] Harboe ZB, Dalby T, Weinberger DM, Benfield T, Mølbak K, Slotved HC, Suppli CH, Konradsen HB, Valentiner-Branth P. Impact of 13-valent pneumococcal conjugate vaccination in invasive pneumococcal disease incidence and mortality. Clin Infect Dis 2014; 59:1066-73; PMID:25034421; http://dx.doi.org/10.1093/cid/ciu524 25034421

[37] Menéndez R, Torres A, España PP, Pérez-Trallero E, López Hontangas JL, Marco F, et al. Pneumococcal serotypes causing community-acquired pneumonia (CAP) among hospitalized adults in Spain using a new urinary antigen detection (UAD) test. The CAPA study Presented at ERS Congress 2014.

[38] Grijalva CG, Wunderink RG, Williams D, Zhu Y, Balk R, Fakhran S, et al. Distribution of pneumococcal serotypes detected through urine analysis among us adults hospitalized with pneumonia after introduction of PCV13. Presented at the 9th International Symposium on Pneumococci and Pneumococcal Diseases, 9–13 March 2014, Hyderabad, India [#ISPPD-0225]

[39] Bonten MJ, Huijts SM, Bolkenbaas M, Webber C, Patterson S, Gault S, van Werkhoven CH, van Deursen AM, Sanders EA, Verheij TJ, et al. Polysaccharide conjugate vaccine against pneumococcal pneumonia in adults. N Engl J Med 2015; 372:1114-25; PMID:25785969 25785969 10.1056/NEJMoa1408544

[40] Tomczyk S, Bennett NM, Stoecker C, Gierke R, Moore MR, Whitney CG, Hadler S, Pilishvili T. Use of 13-valent pneumococcal conjugate vaccine and 23-valent pneumococcal polysaccharide vaccine among adults aged ≥65 years: recommendations of the Advisory Committee on Immunization Practices (ACIP). MMWR Morb Mortal Wkly Rep 2014; 63:822-5; PMID:25233284 25233284 PMC5779453

[41] Huss A, Scott P, Stuck AE, Trotter C, Egger M. Efficacy of pneumococcal vaccination in adults: a meta-analysis. CMAJ 2009; 180:48-58; PMID:19124790 19124790 10.1503/cmaj.080734 PMC2612051

[42] Leventer-Roberts M, Feldman BS, Brufman I, Cohen-Stavi CJ, Hoshen M, Balicer RD. Effectiveness of 23-valent pneumococcal polysaccharide vaccine against invasive disease and hospital-treated pneumonia among people aged 65+: A retrospective case-control study. Clin Infect Dis 2015; 60(10):1472-80; PMID:25669354 25669354 10.1093/cid/civ096

[43] Musher DM. Editorial commentary: should 13-valent protein-conjugate pneumococcal vaccine be used routinely in adults? Clin Infect Dis 2012; 55(2):265-7; PMID:22495544 22495544 10.1093/cid/cis364

[44] Jackson LA, Gurtman A, van Cleeff M, Frenck RW, Treanor J, Jansen KU, Scott DA, Emini EA, Gruber WC, Schmoele-Thoma B. Influence of initial vaccination with 13-valent pneumococcal conjugate vaccine or 23-valent pneumococcal polysaccharide vaccine on anti-pneumococcal responses following subsequent pneumococcal vaccination in adults 50 years and older. Vaccine 2013; 31(35):3594-602; PMID:23688525 23688525 10.1016/j.vaccine.2013.04.084

[45] Pazdiora P Data presented at the 7^th^ Pneumo Surveillance Summit, Istanbul, Turkey, 26-27 9 2014.

[46] Hryniewicz W Surveillance in Poland: BINet experience. Presented at the 7^th^ Pneumo Surveillance Summit, Istanbul, Turkey, 26-27 9 2014.

[47] Maďarová L, Bottková E, Čamajová J, Klement C, Avdičová M Data presented at the 7^th^ Pneumo Surveillance Summit, Istanbul, Turkey, 26-27 9 2014.

[48] Streinu-Cercel A Data presented at the 7^th^ Pneumo Surveillance Summit, Istanbul, Turkey, 26-27 9 2014.

[49] Lobzin YV, et al. Serotypes of Streptococcus pneumoniae causing major pneumococcal infections. J Infectol 2013; 5:36-42.

[50] Mayansky N, Alyabieva N, Ponomarenko O, Lazareva A, Katosova L, Ivanenko A, Kulichenko T, Namazova-Baranova L, Baranov A. Serotypes and antibiotic resistance of non-invasive Streptococcus pneumoniae circulating in pediatric hospitals in Moscow, Russia. Int J Infect Dis 2014; 20:58-62; PMID:24462930 24462930 10.1016/j.ijid.2013.11.005

[51] Kozlov RS, Krechikova OI, Muravyev AA, Mironov KO, Platonov AE, Dunaeva EA, et al. Incidence of community-acquired pneumonia and acute otitis media in children 0-5 years in Russia and role of S. pneumoniae or H. influenzae in the etiology of the diseases. Clin Microbiol Antimicrob Chemother 2013; 15:246-60.

[52] Ceyhan M, Gurler N, Yaman A, Ozturk C, Oksuz L, Ozkan S, Keser M, Salman N, Alhan E, Esel D, et al. Serotypes of Streptococcus pneumoniae isolates from children with invasive pneumococcal disease in Turkey: Baseline evaluation of the introduction of the pneumococcal conjugate vaccine nationwide. Clin Vaccine Immunol 2011; 18:1028-30; PMID:21508171 21508171 10.1128/CVI.00526-10 PMC3122622

[53] Hasçelik AG, Gürler N, Ceyhan M, Öksüz L, Özakın C, Bayramoğlu G, et al. Serotype prevalence and antibiotic resistance among adult invasive Streptococcus pneumoniae isolates in Turkey, 2005–2011. Presented at the 16th Int Society Infect Dis (ICID) 2014 [#40.023].

[54] Kulcsar A Data presented at the 7^th^ Pneumo Surveillance Summit, Istanbul, Turkey, 26-27 9 2014.

[55] World Health Organization (WHO) vaccine-preventable diseases: monitoring system. 2015 global summary. Available at: http://apps.who.int/immunization_monitoring/globalsummary.

[56] Tóthpál A, Dobay O Vaccine coverage of invasive and carried Streptococcus pneumoniae isolates in Hungary. LAM 2014; 24(4):180-5.

[57] Chernyshova LI, Gilfanova AM, Bondarenko AV, Yakimovitch SA, Yanovska VV, Glushkevich TG Streptococcus pneumoniae serotypes distribution in nasopharyngeal carriage in healthy children aged 6 months to 5 years old in Ukraine, 2012–2014. Poster presented at the 7th Pneumo Surveillance Summit, Istanbul, Turkey, 26-27 9 2014.

[58] Skoczyńska A, Kuch A, Sadowy E, Waśko I, Markowska M, Ronkiewicz P, Matynia B, Bojarska A, Wasiak K, Gołębiewska A, et al. Recent trends in epidemiology of invasive pneumococcal disease in Poland. Eur J Clin Microbiol Infect Dis 2015; 34(4):779-87; PMID:25475124 25475124 10.1007/s10096-014-2283-8

[59] National Reference Centre for Pneumococcal Diseases, Slovakia

[60] Stock NK, Maly M, Sebestova H, Orlikova H, Kozakova J, Krizova P. The Czech Surveillance System for Invasive Pneumococcal Disease, 2008-2013: A Follow-Up Assessment and Sensitivity Estimation. PLoS One 2015; 10(6):e0131117; PMID:26125583 26125583 10.1371/journal.pone.0131117 PMC4488342

[61] Tambic Andrasevic A Data presented at the 7th Pneumo Surveillance Summit, Istanbul, Turkey, 26-27 9 2014.

[62] Ceyhan M Hum Vacc Immunotherapeutics 2016; 12:308-13; http://dx.doi.org/10.1080/21645515.2015.1078952 PMC5049732 26325175

[63] Tambic Andrasevic A, Soprek S Antibiotic resistance surveillance in invasive isolates. In: Tambic Andrasevic A, Tambic T, eds. Antibiotic resistance in Croatia. Zagreb: Croatian Academy of Medical Sciences; 2014:119-32.

[64] ESPID Short Oral Presentation Session 1 - Epidemiology and Public Health 14 May 2015; 13:30 15:30, 21.

[65] Yeraliyeva LT, Ramazanova BA, Mustafina KK Developments in national surveillance 2013-2014: Updates on pneumococcal disease from Kazakhstan. Presented at the 7th Pneumo Surveillance Summit, Istanbul, Turkey, 26–27 September 2014.

[66] Kozakova J, Motlova J, Benes C, et al. Invasive pneumococcal disease in the Czech Republic in 2012. Zpravy CEM (SZU, Praha) 2013:22(3):97-104.

[67] Kozakova J, Sebestova H, Krizova P Invasive pneumococcal disease in the Czech Republic in 2013. Zpravy CEM (SZU, Praha) 2014; 23(3):89-97.

[68] Regional Authority of Public Health (RAPH), Banská Bystrica, Slovakia. Available at: www.vzbb.sk.

[69] Tóthpál A, Dobay O Expected clinical effectiveness of PCVs in Hungary. Gyermekgyógyászat 2010; 61:188-91.

[70] Tóthpál A, Dobay O Nasal carriage of Streptococcus pneumoniae among Hungarian children before the wide use of the conjugate vaccine. Acta Microbiologica Et Immunologica Hungarica 2012; 59:107-18; http://dx.doi.org/10.1556/AMicr.59.2012.1.11 22510292

[71] National Institute of Public Health, Romania.

[72] Ceyhan M, Ozsurekci Y, Gürler N, Ozkan S, Sensoy G, Belet N, Hacimustafaoglu M, Celebi S, Keser M, Dinleyici EC, et al. Distribution of Streptococcus pneumoniae serotypes that cause parapneumonic empyema in Turkey. Clin Vaccine Immunol 2013; 20:972-6; PMID:23637041; http://dx.doi.org/10.1128/CVI.00765-12 23637041 PMC3697447

[73] Tirczka T Mikrobiológiai Körlevél 2013No; 3-4. Available at: www.oek.hu/oek.web?to=1303,2129&nid = 104&pid = 3&lang = hun.

[74] Percin D, Altintop YA, Sumerkan B. Ten-year surveillance of invasive Streptococcus pneumoniae isolates in central Turkey prior to the introduction of a conjugate vaccine. J Infect Dev Ctries 2010; 4:560-5; PMID:21045368; http://dx.doi.org/10.3855/jidc.834 21045368

[75] Yalçın I, Gürler N, Alhan E, Yaman A, Turqut M, Celik U, Akçakaya N, Camcioğlu Y, Diren S, Yildirim B Serotype distribution and antibiotic susceptibility of invasive Streptococcus pneumoniae disease isolates from children in Turkey, 2001–2004. Eur J Pediatr 2006; 165(9):654-7; http://dx.doi.org/10.1007/s00431-006-0128-x 16602003

[76] Altun HU, Hascelik G, Gür D, Eser OK. Invasive pneumococci before the introduction of pneumococcal conjugate vaccine in Turkey: antimicrobial susceptibility, serotype distribution, and molecular identification of macrolide resistance. J Chemother 2015; 27:74-9; PMID:24548097; http://dx.doi.org/10.1179/1973947814Y.0000000176 24548097

[77] Ceyhan M, Gürler N, Ozsurekci Y, et al. Pneumococcal serotypes causing childhood invasive pneumococcal disease in turkey, 2011-2012. Presented at the 9th International Symposium on Pneumococci and Pneumococcal Diseases, >9–13 March 2014, Hyderabad, India [#ISPPD-0546].

